# MR-based motion correction for cardiac PET parametric imaging: a simulation study

**DOI:** 10.1186/s40658-017-0200-9

**Published:** 2018-02-01

**Authors:** Rong Guo, Yoann Petibon, Yixin Ma, Georges El Fakhri, Kui Ying, Jinsong Ouyang

**Affiliations:** 1 Department of Engineering Physics, Tsinghua University, Beijing, 10084 China; 2 Key Laboratory of Particle and Radiation Imaging, Ministry of Education, Beijing, 10084 China; 3 Gordon Center for Medical Imaging, Department of Radiology, Massachusetts General Hospital, Boston, MA 02114 USA; 4 Department of Radiology, Harvard Medical School, Boston, MA 02115 USA; 5 Present Address: Department of Electrical and Computer Engineering, University of Illinois at Urbana–Champaign, Urbana, IL 61801 USA; 6 Present Address: Medical Physics Graduate Program, Duke University, Durham, NC 27705 USA

**Keywords:** MR-based PET motion correction, Cardiac PET parametric imaging, PET-MR, Myocardial perfusion

## Abstract

**Background:**

Both cardiac and respiratory motions bias the kinetic parameters measured by dynamic PET. The aim of this study was to perform a realistic positron emission tomography-magnetic resonance (PET-MR) simulation study using 4D XCAT to evaluate the impact of MR-based motion correction on the estimation of PET myocardial kinetic parameters using PET-MR. Dynamic activity distributions were obtained based on a one-tissue compartment model with realistic kinetic parameters and an arterial input function. Realistic proton density/T1/T2 values were also defined for the MRI simulation. Two types of motion patterns, cardiac motion only (CM) and both cardiac and respiratory motions (CRM), were generated. PET sinograms were obtained by the projection of the activity distributions. PET image for each time frame was obtained using static (ST), gated (GA), non-motion-corrected (NMC), and motion-corrected (MC) methods. Voxel-wise unweighted least squares fitting of the dynamic PET data was then performed to obtain *K*_1_ values for each study. For each study, the mean and standard deviation of *K*_1_ values were computed for four regions of interest in the myocardium across 25 noise realizations.

**Results:**

Both cardiac and respiratory motions introduce blurring in the PET parametric images if the motion is not corrected. Conventional cardiac gating is limited by high noise level on parametric images. Dual cardiac and respiratory gating further increases the noise level. In contrast to GA, the MR-based MC method reduces motion blurring in parametric images without increasing noise level. It also improves the myocardial defect delineation as compared to NMC method. Finally, the MR-based MC method yields lower bias and variance in *K*_1_ values than NMC and GA, respectively. The reductions of *K*_1_ bias by MR-based MC are 7.7, 5.1, 15.7, and 29.9% in four selected 0.18-mL myocardial regions of interest, respectively, as compared to NMC for CRM. MR-based MC yields 85.9, 75.3, 71.8, and 95.2% less *K*_1_ standard deviation in the four regions, respectively, as compared to GA for CRM.

**Conclusions:**

This simulation study suggests that the MR-based motion-correction method using PET-MR greatly reduces motion blurring on parametric images and yields less *K*_1_ bias without increasing noise level.

## Background

Dynamic PET imaging is a powerful technique allowing to obtain physiological information using kinetic modeling. Cardiac PET perfusion tracers, including ^13^N-ammonia, ^15^O-water, ^82^Rb, and ^18^F-flurpiridaz, can be used to measure myocardial blood flow (MBF) in order to evaluate the presence and severity of ischemia [1–6]. However, heart motion caused by the pumping action of heart chambers (cardiac motion) and breathing (respiratory motion) can severely blur the PET emission data and generate image artifacts if no motion correction is applied. This can in turn lead to significant bias on the estimation of MBF.

There have been many attempts to develop practical and effective methodology to remove the effects of heart motion [7–11]. Cardiac and/or respiratory gating strategies that “freeze” motion is popular in static PET but have not been effective or successful in dynamic PET because of the substantial noise associated with rejecting a large number of detected PET events in short time frames. Therefore, how to estimate and correct for both cardiac and respiration motions remains an important research topic for cardiac PET imaging. In the past, a number of cardiac/respiratory motion correction methods have been developed for PET. Qiao et al. [12] developed a 4D model that incorporates motion information to produce a motion-free image with all acquired data and evaluated their approach using simulation and phantom studies. Gigengack et al. [13] developed a motion correction method based on dual gating and mass-preserving hyper-elastic imaging registration and evaluated their method on 21 subjects. Lamare et al. [14] evaluated different schemes of combining gated data to obtain motion-free cardiac PET images using dual gated acquisitions; Feng et al. [15] developed dual respiratory and cardiac motion correction methods after, during, and before image reconstruction and evaluated their performance using the Monte Carlo simulation. The emergence of positron emission tomography-magnetic resonance (PET-MR) scanners offers an elegant solution to PET motion correction. A number of MR-based cardiac/respiratory motion correction methods have been developed for cardiac PET. Wang et al. [16] proposed a dual respiratory and cardiac motion correction scheme for myocardial perfusion PET and studied its effectiveness on myocardial perfusion defect detection. Küstner et al. [17] performed respiratory and cardiac PET motion correction with a motion model derived from a simultaneously acquired MR data on 36 subjects. Kolbitsch et al. [18] performed MR-based respiratory and cardiac PET motion correction in simultaneous ^18^F-FDG PET-MR scans of a canine model of myocardial infarct and one human subject. However, none of these studies has evaluated the impact of MR-based cardiac/respiratory motion correction on PET parametric imaging. In this work, the performance of MR-based motion correction was evaluated on parametric myocardial perfusion PET imaging. Realistic PET and MR simulations, in which the true kinetic parameters were known, were performed for such purpose.

## Methods

**Fig. 1:**
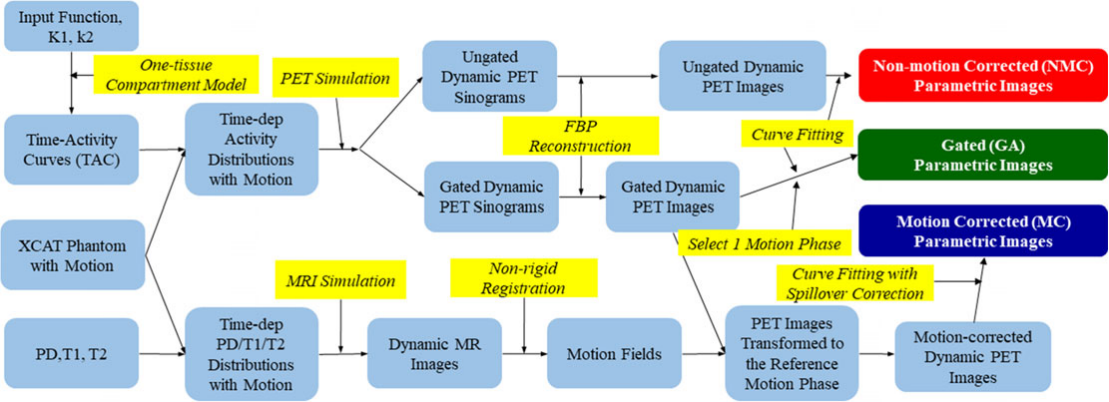
Flowchart of PET-MR data simulation and processing

Figure 1 shows the flowchart depicting the main steps in our study, which include both PET and MR simulations, motion estimation, PET reconstruction, motion correction, and estimation of parametric images.

### PET simulation

PET simulation was performed using the anthropomorphic 4D XCAT phantom [19], which mainly includes heart, lung, liver, and soft-tissue compartments. A non-transmural defect (~ 4 mm across the thickness of the myocardium and ~ 1.2 cm along the myocardial wall) was added to the mid-anterolateral myocardium of the phantom. The simulations were performed to mimic a patient scan on a whole-body Siemens Biograph mMR PET-MR scanner. The PET system has 8 rings of 56 blocks of 8 × 8 lutetium oxyorthosilicate crystals of size 4 × 4 × 20 mm^3^. The PET field of view (FOV) is 59.4 and 25.8 cm in transaxial and axial directions, respectively. The MR system consists of a whole-body superconductive 3-T magnet, an actively shielded whole-body gradient system, and a RF body coil.

**Fig. 2:**
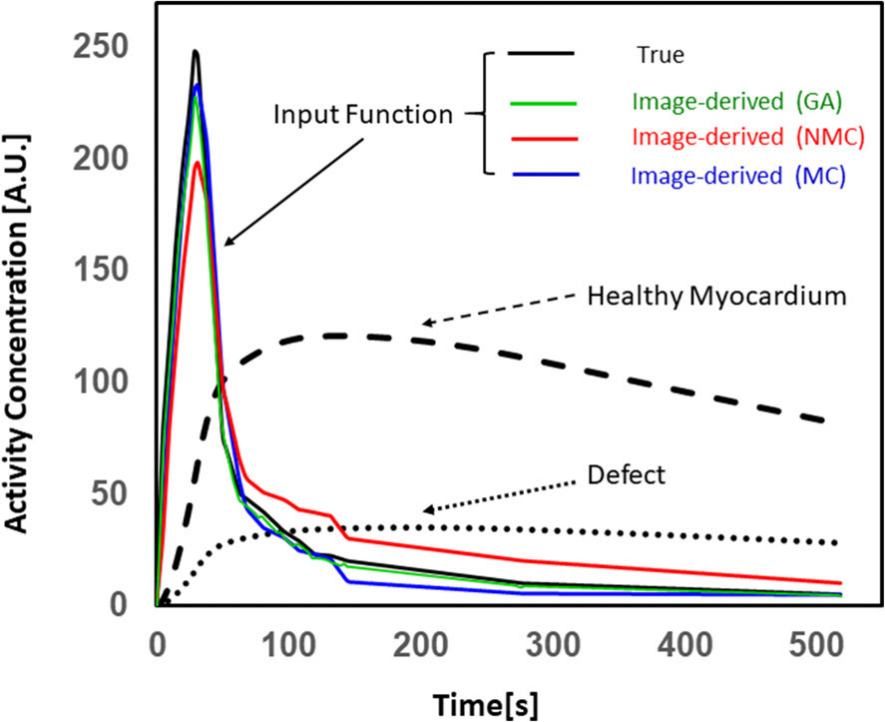
The input function and TACs for the healthy myocardium and defect. The true function is compared with the image-derived input functions using GA, NMC, and MC methods

For the PET simulation, realistic *K*_1_, *k*_2_, and attenuation coefficient values were first assigned for each organ or tissue type. Based on a previously reported ^13^N-ammonia cardiac study [20], we assigned *K*_1_ = 0.80 mL min^−1^ mL^−1^, *k*_2_ = 0.17 min^−1^ to the healthy myocardium and *K*_1_ = 0.36 mL min^−1^ mL^−1^, *k*_2_ = 0.21 min^−1^ to the non-transmural defect. Likewise, an arterial input function (see the true input function in Fig. 2), *C*_*p*_(*t*), was defined based on a previous human ^13^N-ammonia perfusion study [21]. Second, a one-tissue compartment model was used to generate a time-activity curve (TAC) of tissue for each voxel (see Fig. 2 for the TACs of the healthy myocardium and the myocardial defect), ${C}_t^i(t)$, using ${C}_t^i(t)={K}_1^i{C}_p(t)\bigotimes {e}^{-{k}_2^it}$, where *i* is the voxel index. The dynamic (i.e., time-dependent) PET activity distributions were grouped into a series of 8× 5-s, 4 × 10-s, 2 × 20-s, 1 × 40-s, 1 × 2-min, and 1 × 4-min time frames. Third, the motion produced by the XCAT phantom was introduced to generate dynamic PET activity distributions with motion. There are two types of breathing patterns, belly and rib-cage breathing. The former has little impact on the heart motion. Therefore, two different motion studies were performed, one with cardiac motion only (CM) and the other with both cardiac and respiratory motions (CRM). Cardiac motion cycle was evenly divided into ten and five motion phases for CM and CRM, respectively. Respiratory motion cycles were evenly divided into five motion phases. As a result, a total of ten and 25 motion phases were defined, for CM and CRM, respectively. Additionally, a static (ST) study without motion was performed to serve as the reference. Fourth, at each motion phase, a PET forward model, which incorporates gamma ray attenuation and point spread function (PSF) modeling, was used to generate noise-free dynamic sinograms by forward-projection of the dynamic PET activity distributions using:

$$ \overline{\boldsymbol{y}}= AGP\mathbf{x} $$

where **x** = [*x*_1_, *x*_2_, ⋯, *x*_*I*_]^*T*^ contains the activity concentration in all *I* voxels; $\overline{\mathbf{y}}={\left[{\overline{y}}_1,{\overline{y}}_2,\cdots, {\overline{y}}_J\right]}^T$contains the estimated true counts in all *J* sinogram bins; matrix *P* of size *I* × *I* models PSF blurring effects in the image space [full width at half maximum (FWHM) of 4.5 mm was used]; matrix *G* of size *J* × *I* models the geometric forward-projection, which was implemented using Siddon’s ray-tracing method [22]; and diagonal matrix *A* of size *J* × *J* provides the attenuation factor for each sinogram bin. Both scatter and random events were not accounted for in the PET simulation. The sinogram data were scaled so that the total number of true counts in the 4-min time frame, which is the last time frame, is 50,000 for a 3-mm slice. This corresponds to a PET study performed on an adult with an injection dose of about 370–555 MBq. Poisson noise was then added to each sinogram bin based on the mean counts in the bin to generate 25 different noise realizations of sinograms.

### MR simulation

Similar to the PET simulation, realistic proton density (PD) and T1 and T2 parameters (3 T) were assigned to each organ or tissue type for the MRI simulation. The PD (percentage relative to water) and T1 and T2 values are 67% and 830 and 62 ms, respectively, for the healthy myocardium and 77% and 1080 and 82 ms, respectively, for the myocardial defect [23]. Motion from the XCAT phantom was introduced to generate motion-dependent PD/T1/T2 distributions. The MRI simulation in each motion phase was performed using MRiLab [24], an open-source software based on MATLAB. MRI noise, which is due to the signal variations in the receiver chain caused by thermal noise and eddy currents in the imaging object, was not modeled in this study. The standard gradient echo (GRE) sequence was used (TR = 9.56 ms, TE = 2.4 ms, FA = 46°). The simulated MR *k*-space data were then reconstructed to obtain the MR image for each motion phase.

### Motion correction

A reference motion phase was selected for each type of motion, i.e., CM or CRM (end-diastole for CM; end-diastole/end-exhalation for CRM). The motion fields transforming the MR image volume from a given motion phase to the reference motion phase were obtained by applying a non-rigid image registration technique, which is based on the Demons algorithm [25–27].

Four different studies using static (ST), gated (GA), non-motion-corrected (NMC), and motion-corrected (MC) data were performed. For the ST study, the PET data, in which neither motion nor noise was introduced, were reconstructed using filtered back-projection (FBP) for each time frame. For the GA study, only the data corresponding to the reference motion phase were reconstructed using FBP. For the NMC study, the PET data generated in each motion phase were first reconstructed using FBP in each time frame. The resulting PET images across all the motion phases were then summed for the time frame. For the ST, GA, and NMC studies, the attenuation map in the reference motion phase was used during the PET reconstruction. For the MC study, the attenuation map for a given motion phase was obtained by transforming the reference attenuation map to the motion phase using the corresponding motion fields measured by MRI. The reconstructed PET image by FBP for each motion phase was transformed to the reference motion phase using the motion fields measured by MRI in each time frame. The final motion-corrected PET image in the time frame was then obtained by summing up all the transformed PET images (including the one in the reference motion phase). Because the goal of our study is to assess only the impact of motion correction (including correction on both the emission data and attenuation map) on dynamic PET, we used the same attenuation map, which was generated by the XCAT phantom, for both the simulations and reconstructions.

### Estimation of kinetic parameters

For each study, a myocardium mask was created by applying a threshold to the static PET image reconstructed from the 4-min time frame (the last time frame). To account for the spillover effects from both the left ventricle (LV) and right ventricle (RV) blood pools, the measured myocardial activity concentration in time frame *t*_*m*_ can be modeled as:

$$ {C}_{\mathrm{PET}}^i\left({t}_m\right)={f}_{\mathrm{LV}}^i.{C}_{\mathrm{LV}}\left({t}_m\right)+{f}_{\mathrm{RV}}^i.{C}_{\mathrm{RV}}\left({t}_m\right)+\left(1-{f}_{\mathrm{LV}}^i-{f}_{\mathrm{RV}}^i\right)\frac{1}{\Delta {t}_m}{\int}_{\Delta {t}_m}{C}_t^i\left(\tau \right) d\tau $$

where *C*_LV_(*t*_*m*_) (*C*_RV_(*t*_*m*_)) is the LV (RV) TAC, ${f}_{\mathrm{LV}}^i$ (${f}_{\mathrm{RV}}^i$) is the fractional spillover from LV (RV) in voxel *i* that accounts for the contamination of myocardial TACs by activity from the ventricle blood polls due to the limited PET spatial resolution, and *∆t*_*m*_ is the duration of the time frame. Afterwards, voxel-wise *K*_1_ values were estimated within the myocardium mask by unweighted least squares curve-fitting of the reconstructed myocardial PET TACs [i.e.,${C}_{\mathrm{PET}}^i\left({t}_m\right)$] using MATLAB CFTOOL. We defined four 0.18-mL regions of interest (ROIs) at different locations in the myocardium. Each ROI was carefully chosen to be away from the edges of the true myocardium. For each ROI and each method (i.e., ST, GA, NMC, and MC), the average *K*_1_ value was first computed for each noise realization. The mean (${\overline{K}}_1$) and standard deviation (${\sigma}_{K_1}$) of *K*_1_ values were then computed across all the noise realizations using:

$$ {\overline{K}}_1=\frac{1}{N}\sum \limits_n{K}_1^n,\kern1em {\sigma}_{K_1}=\sqrt{\frac{1}{N-1}\sum \limits_n{\left({K}_1^n-{\overline{K}}_1\right)}^2}, $$

where ${K}_1^n$ is the average *K*_1_ value within the ROI for noise realization *n* and *N* is the total number of noise realizations. The performance of MR-based PET motion correction for parametric myocardial perfusion PET imaging was evaluated using the ST results as the gold standard for each ROI. For method M (i.e., GA, NMC, or MC), the bias (${b}_{K_1}^{\mathrm{M}}\Big)\kern0.5em$was computed using:

$$ {b}_{K_1}^{\mathrm{M}}=\frac{1}{{\overline{K}}_1^{\mathrm{ST}}}\left({\overline{K}}_1^{\mathrm{M}}-{\overline{K}}_1^{\mathrm{ST}}\right), $$

where ${\overline{K}}_1^{\mathrm{ST}}$ and ${\overline{K}}_1^{\mathrm{M}}$ are the mean *K*_1_ values for ST and method M*,* respectively. The standard deviation reduction of MC relative to GA was computed using:

$$ {\delta}_{K_1}^{\mathrm{MC}}=\left|{\sigma}_{K_1}^{\mathrm{MC}}-{\sigma}_{K_1}^{\mathrm{GA}}\right|/{\sigma}_{K_1}^{\mathrm{GA}}, $$

where ${\sigma}_{K_1}^{\mathrm{MC}}$ and ${\sigma}_{K_1}^{\mathrm{GA}}$ are the standard deviation of *K*_1_ values for MC and GA, respectively.

### Motion estimation based on a human study

In order to demonstrate the feasibility of using MRI to estimate both the cardiac and respiratory motion fields of the heart, a subject was scanned on a Siemens 3-T system using a fast low angle shot (FLASH) MRI sequence with golden-angle based radial sampling of the *k*-space (denoted hereinafter as Rad-FLASH) [28]. The sequence also acquired a fast slice-projection navigator (NAV) echo every TR to track the diaphragm position. The subject was instructed to breath freely during the scan. The total imaging time was about 5 min. Twenty coronal Rad-FLASH slices were acquired to cover the whole thorax. The acquisition parameters were slice thickness = 8 mm, TE = 1.5 ms, TR = 70 ms, FA = 30°, FOV = 320 × 320 mm^2^, and the total number of radial *k*-lines acquired per slice = 5120. Moreover, an electrocardiogram (ECG) was used to monitor the cardiac cycle. For the processing of Rad-FLASH data, we first extracted the subject’s diaphragm position using the NAV signal. We then assigned a respiratory phase (out of total of five phases) and a cardiac phase (out of total of six phases) to each acquired radial *k*-line (spoke) based on amplitude bins defined within the moving range of the diaphragm and on the time delay relative to the R-wave from the ECG, respectively. The spokes in all the motion phases were reconstructed simultaneously using kt-FOCUSS [29], which is a compressed sensing technique. This procedure allowed obtaining one MR image volume for each one of the 30 motion phases. Non-rigid registration of the MR volumes was then used to estimate the motion fields between each motion phase and the reference one, which was chosen to be the end-exhalation/end-diastolic phase. For CM, only the motion phases at the end of exhalation were used.

## Results

**Fig. 3:**
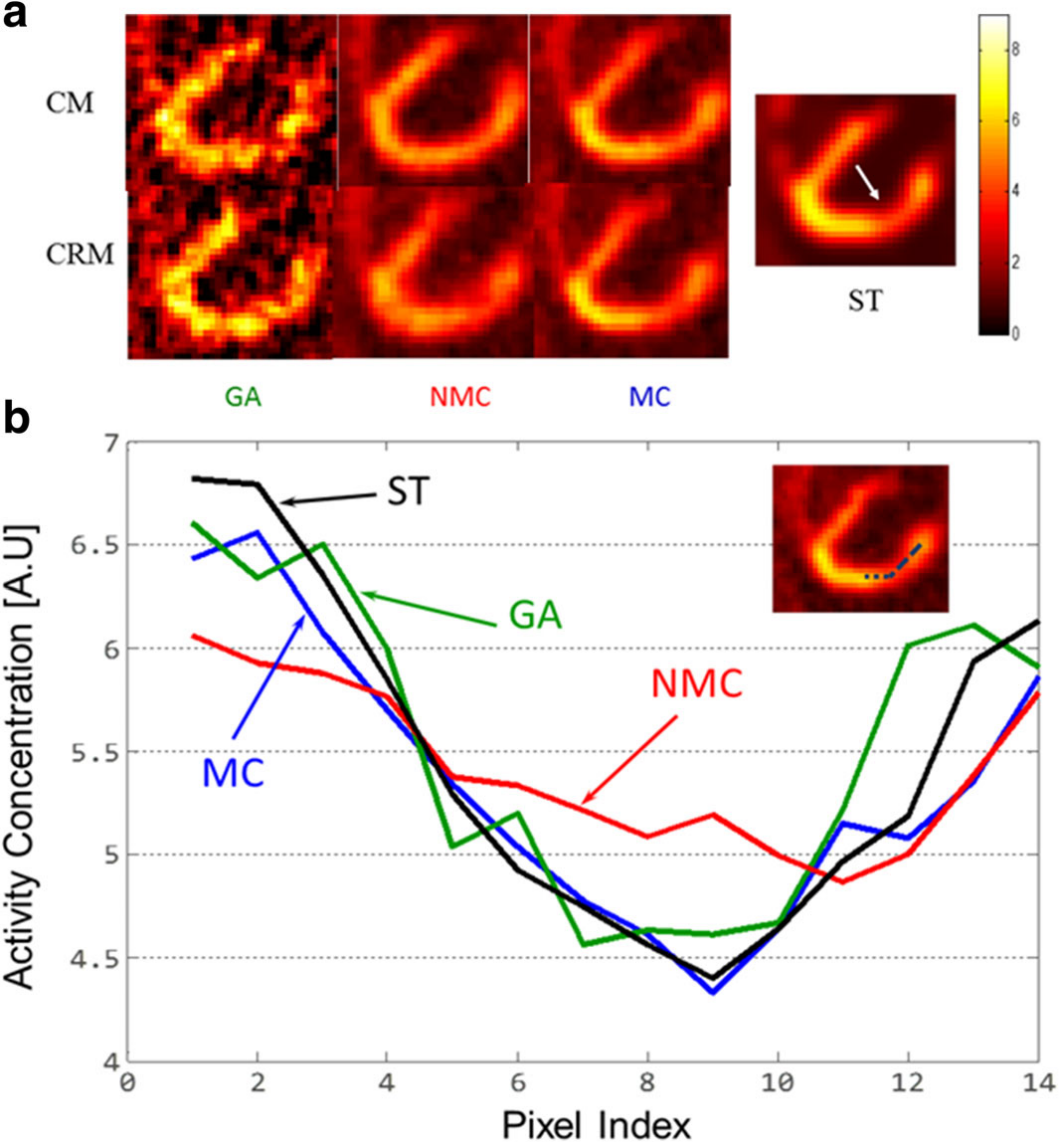
Reconstructed PET images and line profiles. **a** Reconstructed GA, NMC, and MC PET images for CM and CRM as well as reconstructed ST PET image. All the images were obtained using the data acquired from four to ten minutes after the injection. The GA, NMC, and MC images are for one noise realization. The arrow on the ST image points to the defect. **b** GA, NMC, MC line profiles (for CRM) as well as ST line profile. The profiles were drawn along a line (shown on the image at the top-right corner) connecting the anterobasal and apical regions and going through the center of the defect

For both CM and CRM, Fig. 3 a shows the same PET slice through the myocardial defect using the data generated from 6 to 10 min after the tracer injection for one noise realization (The ST image was obtained using the noise-free data). The noise levels are high in the GA images because only 10 and 4% of all the detected counts were used in PET reconstruction for CM and CRM, respectively. The NMC images have less noise but are blurred by motion. As compared to the ST image, the blurring effect results in a thicker myocardium wall in the NMC images. CRM causes more motion blurring than CM. The MC images have greatly reduced motion artifact and similar noise level as compared to the NMC images.

Figure 3b shows the activity concentration profiles along a line connecting anterobasal and apical regions through the center of the defect for CRM. GA and MC have similar profiles to ST except that the GA profile has more fluctuations due to high noise level in the GA image. For NMC, the contrast between the defect and the healthy myocardium is much lower as compared to ST, GA, and MC.

**Fig. 4:**
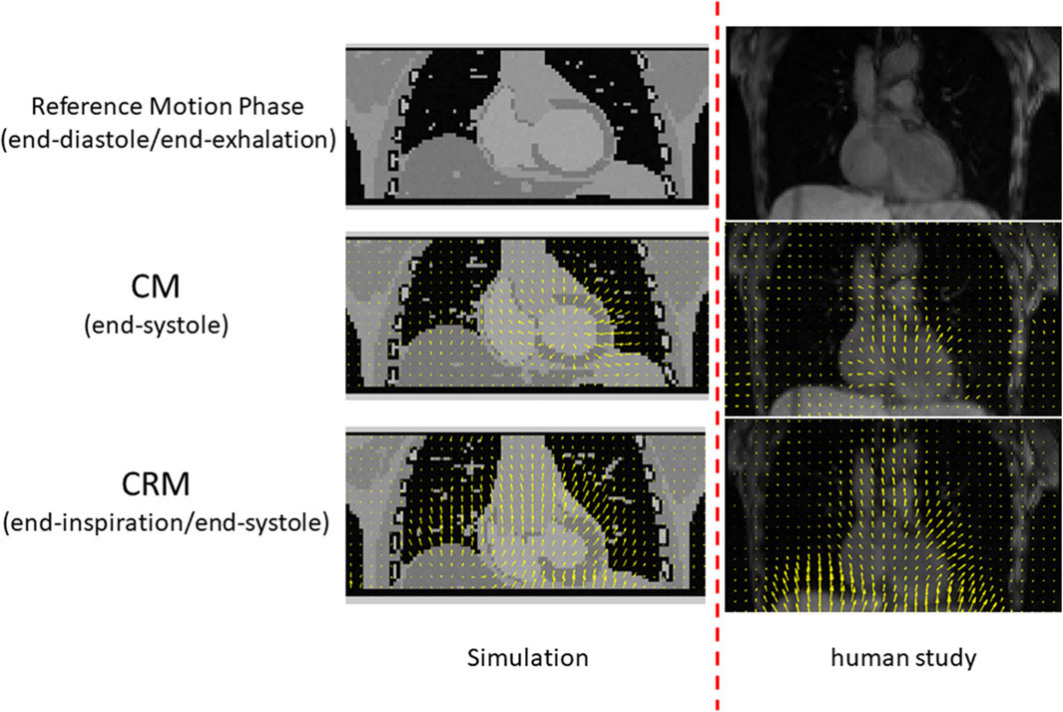
Coronal MR images for two different motion phases along with the estimated motion fields between the two motion phases

Figure 4 shows the coronal MR images in two different motion phases together with the motion fields estimated between the two motion phases for both CM and CRM. Most motion can be seen in the heart region and entire torso for CM and CRM, respectively. Similar motion fields can be found for both the simulation and the human study.

**Fig. 5:**
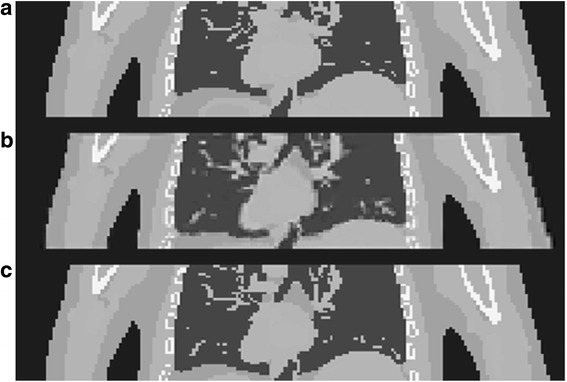
Motion phase-dependent PET attenuation maps. The attenuation map in the reference motion phase was transformed to end-inspiration/end-systole using the estimated motion fields. The resulting attenuation map is similar to the true attenuation map for end-inspiration/end-systole obtained directly from XCAT. **a** Reference motion phase (end-diastole/end-exhalation) **b** Transformed from the reference phase to end-inspiration/end-systole **c** End-inspiration/end-systole (directlyu form XCAT)

Figure 5 shows the coronal attenuation map in the reference motion phase (end-diastole/end-exhalation) (Fig. 5a); the attenuation map transformed from the reference phase to the phase of end-inspiration/end-systole using the motion fields measured by MRI (Fig. 5b) and the true attenuation map generated by the XCAT phantom in the phase of end-inspiration/end-systole (Fig. 5c). For the phase of end-inspiration/end-systole, the transformed attenuation map (Fig. 5b), which was obtained using the motion fields measured by MRI, matches well with the true attenuation map (Fig. 5c).

**Fig. 6:**
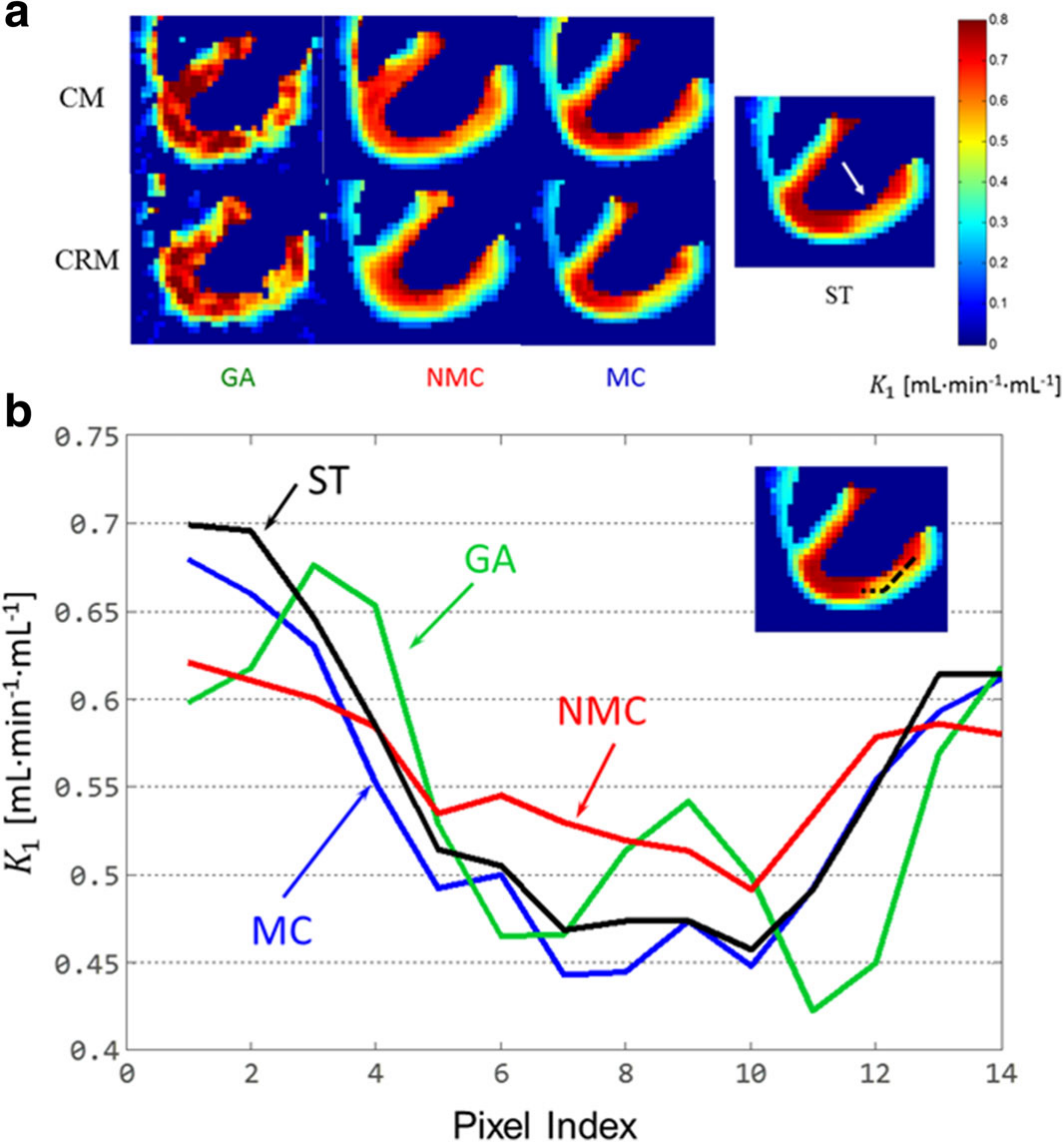
Estimated *K*_1_ maps and line profiles. **a** GA, NMC, and MC *K*_1_ maps for CM and CRM as well as ST *K*_1_ map. The GA, NMC, and MC *K*_1_ maps are for one noise realization. The arrow on the ST map points to the defect. **b** GA, NMC, MC line profiles (for CRM) as well as ST line profile. The profiles were drawn along a line (shown on the map at the top-right corner) connecting the anterobasal and apical regions and going through the center of the defect

For one of the noise realizations, Fig. 6a shows the same *K*_1_ slice through the myocardial defect for both CM and CRM. Similar to Fig. 3a, the noise level is high for GA. The NMC *K*_1_ maps have less noise but depict high motion blurring. The MC *K*_1_ maps have greatly reduced motion artifacts and a noise level similar to that of the NMC maps.

Figure 6b shows the *K*_1_ profiles along a line connecting anterobasal and apical regions through the center of the defect for CRM. The GA and ST profiles match well even though the GA profile has more fluctuation. The MC profile matches well with the ones for ST and GA but with much lower fluctuations. However, the NMC profile substantially deviates from the ST profile.

**Fig. 7:**
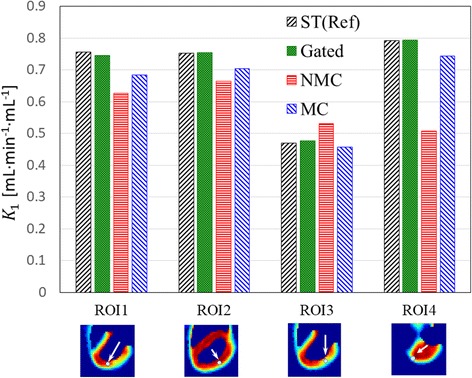
Mean *K*_1_values estimated from 25 noise realizations. Each white arrow and a small circle were only used to indicate the approximate location of the ROI. Please see the text in the “Methods” section for the details on how the ROIs were defined

**Fig. 8:**
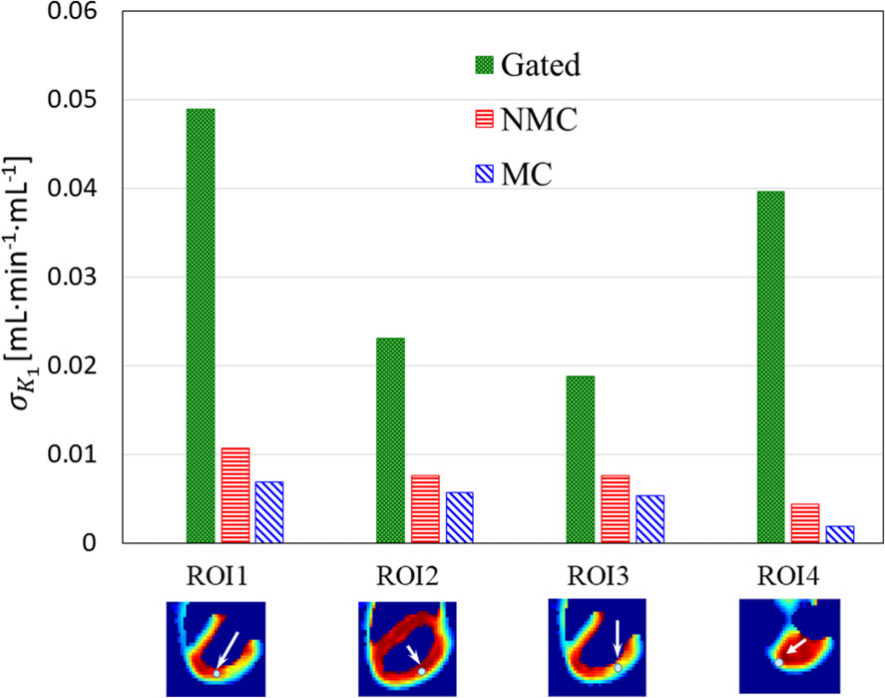
Standard deviation of *K*_1_ values estimated from 25 noise realizations. Each white arrow together with a small circle were only used to indicate the approximate location of the ROI. Please see the text in the “Methods” section for the details on how the ROIs were defined

Finally, Figs. 7 and 8 show the mean and standard deviation of *K*_1_ values for the four selected ROIs computed from the 25 noise realizations for CRM. For all the four ROIs, the MC bias is less than that for NMC using ST as the gold standard. MC yields an absolute reduction of *K*_1_bias, which was computed as the percentage of average *K*_1_using ST, by 7.7, 5.1, 15.7, and 29.9% in ROIs 1, 2, 3, and 4, respectively, as compared to NMC. As compared to the ground truth *K*_1_ values used for simulating the PET dynamic data, ST, GA, NMC, and MC yielded “true bias” (i.e., percentage relative to the ground truth) of − 5.4, − 6.9, − 21.6, and −14.3%, respectively, for ROI1; − 5.8, − 5.8, − 16.8, and − 12.0%, respectively, for ROI2; 30.6, 32.4, 47.6, and 27.0%, respectively, for ROI3; and − 0.9, − 0.7, − 36.6, and − 7.0%, respectively, for ROI4. The true bias values are particularly high for ROI3, which is the defect region, because of activity spillover due to PET partial volume effect. The greatest improvement in PET quantitation by MC as compared to NMC was seen in ROI4 where the motion was the biggest. As expected, GA yields much higher variance than both NMC and MC because MC uses all the PET counts in the PET reconstruction, while GA uses only a fraction of the PET counts. The *K*_1_ standard deviation reductions by MC relative to GA are 85.9, 75.3, 71.8, and 95.2% in the four ROIs, respectively, for CRM.

## Discussion

Comparing NMC to MC, we expect MC to have less bias than NMC. Furthermore, because the number of coincidence events used in NMC and MC was the same, we expect MC and NMC to yield similar variance. These expectations are consistent with the results obtained in this study. For both static and parametric images/profiles as shown in Figs. 3 and 6, respectively, we found that MC has less bias than NMC using ST as the reference while the noise levels for NMC and MC are similar. Similar results were also found in the ROI studies as shown in Figs. 7 and 8.

Comparing GA to MC, we expect MC to have higher bias than GA because MR-based motion correction will not be perfect. Also, we expect NMC to have much lower variance than GA because MC uses all the coincidence events, while GA uses only a fraction of the coincidence events. These expectations are again consistent with our results. As shown in Figs. 3, 6, 7, and 8, MC has much lower noise level than GA. If we use ST as the reference, MC has higher bias than GA.

We have also noticed that due to lower amplitude of the motion, cardiac motion yields less blurring than respiratory motion. However, motion correction for cardiac motion still plays a role. As shown in Figs. 3a and 6a, the myocardium wall becomes noticeably thinner if motion correction is applied.

In this study, the motion fields used for PET motion correction were derived from simulated MR data. However, it is always questionable whether accurate motion fields can be estimated using real data. In Fig. 4, we show in a human MR study that a 5-min golden-angle radial MR acquisition used along with compressed sensing image reconstruction (kt-FOCUSS [29]) can generate 4D MR images with good quality enabling estimation of both cardiac and respiratory motion fields. We found both the simulation and the human study produced similar motion fields. Of course, it is highly desirable to keep the MR acquisition for motion measurement as short as possible so that more imaging time can be dedicated to clinical MR sequences. This can be achieved using low-rank reconstruction technique [30], which takes advantage of the spatiotemporal correlation among the motion phases using compressed sensing [29, 31]. The imaging time can be further reduced using parallel imaging techniques [28, 32].

This study is mainly dedicated to the evaluation of MR-based motion correction on parametric PET imaging. Accurate estimation of kinetic parameters, such as MBF, is challenging because of the high noise levels present in short dynamic frames. This problem becomes more severe if cardiac or dual cardiac/respiratory gating is used because a large portion of data is discarded during reconstruction. This study shows that we are capable of making high quality parametric PET images using MR-based motion correction.

In this study, we used FBP along with post-reconstruction motion correction rather than iterative reconstruction algorithms to obtain dynamic images because it is well known that iterative reconstruction algorithms, such as OSEM, lead to spatially variant spatial resolution and noise characteristics. For dynamic PET, spatially variant spatial resolution across different time frames can lead to errors on the estimated kinetic parameters.

In this study, the input function was assumed to be obtained by an arterial blood sampling, which is challenging in practice. Image-derived input function is an elegant and attractive noninvasive alternative to blood sampling [33, 34]. For cardiac perfusion PET, the image-derived input function can be obtained by computing activity concentration within an ROI within the blood pool of the left ventricle. Such method is practical for cardiac PET because the heart is within the FOV. However, if NMC PET images are used for such purpose, errors may be introduced into the input function due to motion. We defined an ROI of ~ 4 pixels in the blood pool (the ROI was kept away from the myocardium to avoid the spillover effect) and obtained image-derived input functions using GA, NMC, and MC methods. Figure 2 shows that the image-derived input functions using GA and MC agree well with the true input function, while NMC yields large errors. Therefore, image-derived input functions should be obtained using GA or MC methods.

In this study, an assumption that each PET event can be correctly assigned to a motion phase was made. In a real PET-MR scan, motion phase must be tracked whenever PET data are acquired in order to perform MR-based PET motion correction. Cardiac motion phases can be tracked by the relative delay to R wave using ECG [35] or PET list-mode data. Respiratory motion phases can be tracked by either bellows, a pencil-beam MR navigator through diaphragm [35], or PET list-mode data. [36] Although simultaneous PET-MR is ideal for MR-based PET motion correction, it is still feasible to use sequential PET-MR for the purpose if no body motion occurs between the PET and MRI scans. However, for sequential PET-MR, external motion-phase-tracking devices, such as ECG and bellows rather than MR-based motion-phase-tracking methods must be used. Even for simultaneous PET-MR, if body motion occurs during the scan, motion fields must be either re-measured or corrected.

In this study, cardiac and respiratory cycles were binned into multiple motion phases. In a real human scan, MR *k*-space data used for motion estimation for a given motion phase are acquired only when the motion phase is reached. Many cardiac and/or respiratory cycles are needed to collect enough *k*-space data so that MR images with good image quality can be reconstructed. This implies that long MR imaging time is required for the motion measurement. Moreover, the internal motion of the myocardium may not be measured correctly if its MR signals appear uniform using a standard GRE sequence. Applying tagging in three different directions is a solution to such problem but requires much more imaging time [37, 38].

The XCAT phantom can be used to generate a true attenuation map for each motion phase. However, in order to mimic a real PET-MR scan, the attenuation map used in the PET reconstruction in a given motion phase other than the reference phase was not generated by the XCAT phantom for the MC study. Instead, the reference attenuation map was transformed using the motion fields measured by MRI to obtain the motion phase-dependent attenuation maps. Because the purpose of this study is to assess the performance of MR-based motion correction on PET parametric imaging, the attenuation map in the reference motion phase was directly derived from the XCAT phantom. In a real PET-MR study, however, the currently implemented method for PET attenuation correction is to segment tissues into a few different types based on MR images using Dixon sequence and then assign a single attenuation coefficient to each tissue type [39].

In the future, in vivo animal and human PET-MR studies will be performed to further assess the performance of MR-based motion correction for PET parametric imaging. Because the true kinetic parameters are unknown for in vivo studies, gated results with high statistics can be used as the silver standard for the assessment.

## Conclusions

In this paper, a simulation study was performed to evaluate the performance of MR-based motion correction on parametric myocardial perfusion PET imaging using PET-MR. The heart motion on PET images was corrected using motion fields extracted from MRI images. Such correction was then evaluated on the estimation of *K*_1_ values. Our results show that the MR-based motion correction method removes motion blurring and results in less bias on estimated *K*_1_ values than the non-motion correction method. As compared to the conventional gating method, the MR-based motion correction method results in slightly higher or similar bias but much lower variance on the estimated *K*_1_ values. We have also demonstrated from a human MR study that both cardiac and respiratory motion fields can be estimated using a single MR sequence that incorporates both cardiac and respiratory motion tracking.

## Abbreviations

3 T: 3 tesla
CM: Cardiac motion only
CRM: Both cardiac and respiratory motions
CT: Computed tomography
ECG: Electrocardiogram
FA: Flip angle
FBP: Filtered back-projection
FLASH: Fast low angle shot
FOCUSS: Focal underdetermined system solver
FOV: Field of view
FWHM: Full width at half maximum
GA: Gated
GRE: Gradient echo
LV: Left ventricle
MATLAB: Matrix laboratory
MBF: Myocardial blood flow
MC: Motion-corrected
MR: Magnetic resonance
MRI: Magnetic resonance imaging
NAV: Navigator
NMC: Non-motion-corrected
PD: Proton density
PET: Positron emission tomography
PSF: Point spread function
Rad-FLASH: Radial FLASH
ROI: Region of interest
RV: Right ventricle
ST: Static
TAC: Time-activity curve
TE: Echo time
TR: Time of repetition
XCAT: Extended cardiac-torso
